# Evaluating the Technical and Sustainable Viability of Dental Floss Production From Chambira

**DOI:** 10.7759/cureus.97162

**Published:** 2025-11-18

**Authors:** Carlos Espinoza, Carlos Albán, Silvia Reinoso, Blanca Badillo, Andrea C Merino, Karla Arellano

**Affiliations:** 1 School of Dentistry, Universidad Nacional de Chimborazo, Riobamba, ECU; 2 Pediatric Dentistry, Universidad Hemisferios, Riobamba, ECU; 3 Pharmacology, Mundo Farma, Riobamba, ECU

**Keywords:** astrocaryum chambira, community oral health, dental floss, environmental health, sustainability

## Abstract

Aim: This study addressed the growing concern about the environmental and health impact of synthetic dental floss by exploring an ecological and sustainable alternative for oral hygiene in low-income communities. The main objective was to evaluate the technical and sustainable viability of dental floss derived from the Chambira palm (*Astrocaryum chambira*), a species native to the Ecuadorian Amazon, for its possible integration into an oral health project aimed at the Waorani nationality.

Methodology: A methodology was designed for the artisanal production of the Chambira dental floss, which was subjected to a rigorous comparative analysis with commercial products. To determine its potential, technical and sustainable viability models were used, evaluating economic, social, and ecological aspects.

Results: The results showed that the Chambira floss presents physical and mechanical characteristics comparable to those of commercial dental floss. The technical viability analysis revealed a moderate production cost and a public acceptance rate of 81%. For its part, the sustainability study confirmed a positive relationship between the responsible use of the resource and the conservation of the species.

Conclusions: This study establishes that the production of Chambira-based dental floss is not only technically feasible but also commercially sustainable. The proposal promotes an ecological and environmentally friendly product, capable of contributing to the oral health of local populations without compromising the natural habitat of the palm.

## Introduction

Oral health is a fundamental pillar of overall well-being. The use of dental floss is universally recognized as the most effective tool for the mechanical removal of bacterial plaque and food debris from interdental spaces, areas often inaccessible to the toothbrush [1]. However, the vast majority of commercially available dental floss products are composed of synthetic materials derived from polymers (such as nylon, Teflon, or polyethylene), whose production contributes significantly to environmental pollution and whose disposal presents a serious challenge due to their non-biodegradability [2,3].

This problem is aggravated when considering its impact on communities with limited access to health services and environmental awareness, where the dependence on inexpensive but polluting products is greater [4,5,6]. Consequently, there is a critical need to develop ecological and sustainable alternatives that maintain the required mechanical efficacy, minimize environmental impact, and are accessible for focused public health projects [7,8]. This investigation is conducted within the framework of a broader institutional project focused on developing sustainable oral hygiene solutions for vulnerable Amazonian communities [9].

In response to this environmental and social imperative, the current investigation focuses on the Chambira palm (*Astrocaryum chambira*), a species native to the Ecuadorian Amazon. This plant is known for its natural fibers, which possess exceptional tensile strength and flexibility, characteristics that position it as a viable candidate for the artisanal production of dental floss [10,11,12]. Chambira not only offers a renewable and biodegradable resource but also promotes the revaluation of ancestral knowledge [13,14] and generates potential for local economic development [15,16], aligning with the principles of a circular and fair economy [17,18].

The preliminary findings and methodological foundation for this article originated from the thesis entitled "Evaluation of the technical and sustainable feasibility of producing dental floss based on Chambira," authored by Córdova Guerrero and tutored by Espinoza Chávez from the National University of Chimborazo (UNACH), which served as the essential starting point and foundation for the present expanded research [19].

The main objective of this study was to evaluate the technical, mechanical, and sustainable viability of dental floss production using fiber from the Chambira palm.

To achieve this central objective, the following specific and measurable objectives were established:

(1) To quantify and compare the mechanical properties (tensile strength, elasticity, abrasion resistance, and flexural resistance) of the waxed Chambira dental floss against commercial dental flosses (silk and synthetic), using the descriptive analysis mean ± standard deviation (SD) to validate its functional performance.

(2) To determine the technical viability through the detailed description of the artisanal formulation process, the calculation of fiber yield, and the standardization of the physical properties of the final product.

(3) To evaluate the sustainable viability using the triple bottom line (TBL) model, quantifying the unit production cost (economic) and public acceptance of the product (social), and confirming the ecological sustainability of the fiber collection method.

It is hypothesized that dental floss made with Chambira fiber, once treated with natural wax, will exhibit statistically comparable mechanical and performance properties (tensile strength and elongation) to commercial silk dental floss, thereby justifying its technical viability as an ecological and sustainable alternative.

## Materials and methods

Study design and experimental conditions

This study used an experimental, comparative, and descriptive design. The investigation was based on the artisanal production of Chambira dental floss and its subsequent rigorous comparison with two commercial dental flosses. All mechanical tests were conducted under controlled laboratory conditions: ambient temperature of 22 ± 2 °C and relative humidity of 50% ± 5%.

Material acquisition and preparation

Population and Sampling

The population consisted of fibers obtained from the leaves of the Chambira palm (*A. chambira*), collected in the province of Pastaza, Ecuador. Intentional convenience sampling was used, selecting only fibers from young, healthy plants.

Fiber Preparation

Fibers were obtained via a manual defibering process, washed with distilled water, and air-dried in controlled shade for 24 hours. For the testing phase, only filaments with a minimum length of 1.5 m were selected.

This study was conducted with a descriptive, qualitative, and cross-sectional approach. Its design was based on an experimental-type investigation, as it relied on the direct observation of the dental floss manufacturing process and the selection of the sample that presented the most optimal characteristics for its use.

Formulation and waxing process

The formulation used for the final Chambira dental floss (referred to as *Waxed Chambira Floss*) was as follows:

Ingredients

Thin Chambira fibers, 41 g of coconut oil wax, 60 g of natural beeswax, 30 g of soluble gum Arabic, and 0.25 mL of natural mint flavoring.

Process

The wax mixture was heated in a borosilicate beaker until it reached a stable immersion temperature of 70 °C ± 5 °C. The Chambira filaments were submerged and quickly drawn through the liquid wax, performing two rapid passes per segment to ensure a uniform and light coating, replicating the texture of commercial flosses.

Drying

The waxed floss was left to rest for 30 minutes for complete hardening

Comparative mechanical testing protocol

For each mechanical test (Table 1), a sample size of *n* = 3 independent repetitions was used for each type of dental floss (Untreated Chambira, Waxed Chambira, "Cepimax" commercial dental floss (Acromax Laboratories, Quito, Ecuador), and "Colgate Total" commercial dental floss (Colgate-Palmolive Company, New York)).

**Table 1:** Mechanical testing protocol.

Test	Objective and equipment	Procedure	Sample length
Diameter Quantification	To determine the diameter for tensile strength calculation. Equipment: Carl Zeiss Stemi508 (Carl Zeiss Microscopy GmbH, Göttingen, Germany) stereo microscope	Five measurements were taken along each segment to obtain the average.	10 cm
Tensile Strength	To evaluate the maximum force the floss withstands before breaking. Equipment: An experimental setup with calibrated weights and a fixed support	A controlled progressive load was applied until the floss failed.	10 cm (initial clamping length)
Elasticity and Elongation	To measure the percentage of stretching without breaking. Equipment: Calibrated weights and a high-precision millimeter ruler	The maximum elongation of the floss was measured under a fixed load.	20 cm (initial clamping length)
Abrasion Resistance	To simulate interdental friction wear. Equipment: Experimental setup with acrylic resin crown replicas and weights	The floss, under constant tension (200 g), was subjected to controlled back-and-forth movements. The number of cycles until rupture was counted.	45 cm

Sustainable viability analysis

Sustainable viability was assessed using the triple bottom line (TBL) model, divided into three dimensions:

(1) Economic: Calculation of the unit production cost of the waxed Chambira floss

(2) Social: Analysis of public acceptance through a descriptive survey applied to a sample of 227 individuals, using frequency and percentage analysis. Appendix A provides the detailed survey instrument.

(3) Ecological: Confirmation that the collection method used does not compromise the regeneration of the Chambira palm

Statistical analysis

All quantitative data obtained from the mechanical tests were analyzed using descriptive statistics. Results are presented as the mean ± standard deviation (SD) of three independent replicates for each parameter, reflecting the precision and variability of the measurements in accordance with scientific publishing standards.

## Results

The research results are presented in three subsections corresponding to the study’s specific objectives: the evaluation of technical viability (physical and mechanical properties) and sustainable viability (TBL model). All quantitative data are reported as the mean ± SD from three independent replicates (*n* = 3).

Technical viability evaluation: physical properties

Floss Diameter

Morphological analysis using a calibrated optical microscope revealed that the untreated Chambira dental floss had an initial diameter of approximately 0.9 mm. After the waxing process, the average diameter slightly increased to 1.17 ± 0.05 mm. This diameter is considered suitable for interdental cleaning functionality. The natural wax coating proved to be uniform, which is essential to prevent fraying during use.

Comparative mechanical testing protocol results

The evaluation results of the mechanical properties of the Waxed Chambira floss compared to commercial flosses (Silk and Dental floss) and the untreated floss are detailed in Table 2.

**Table 2:** Comparison of mechanical properties of evaluated dental flosses (mean ± SD, n = 3). Tensile strength: The values are expressed as stress (MPa = N/m²), converted from the maximum force (kgF). The conversion was performed using the mean cross-sectional area of the waxed Chambira floss (1.075 × 10⁻⁶ m²) to allow for comparative analysis. Elasticity: Elongation in millimeters Stretching with weight: % Flexural resistance: number of folds Abrasion resistance: number of frictions

Test type	Untreated Chambira floss	Waxed Chambira floss	Commercial silk floss	Commercial synthetic floss
Tensile Strength	5.5 ± 0.5	10.9 ± 0.9	13.7 ± 1.4	18.2±1.7
Elasticity	20.0±1.5	24.0 ± 2.0	24.0 ± 2.0	26.0 ± 2.5
Stretching with Weight	5.0 ± 0.5	15.0 ± 1.5	15.0 ± 1.5	30.0 ± 2.0
Flexural Resistance	125 ± 15	300 ± 30	360 ± 35	650 ± 50
Abrasion Resistance	35 ± 5	65 ± 8	100 ± 12	200 ± 20

Tensile Strength

Tensile strength is presented as the maximum stress (MegaPascals (MPa)) that the floss withstands before breaking, a critical measure of the material's ability to handle tension during use. The Waxed Chambira floss registered a mean stress of 10.9 ± 0.9 MPa. This value is 100% higher than the untreated Chambira floss (5.5 ± 0.5 MPa), demonstrating the positive impact of waxing. Although the stress supported is lower than that of commercial silk floss (13.7 ± 1.4 MPa) and synthetic floss (18.2 ± 1.7 MPa), the value obtained falls within a functional range.

Elasticity and Elongation

The elasticity test, measured on 20 cm segments, showed that the Waxed Chambira floss exhibited a mean elongation of 24.0 ± 2.0 mm, identical to the mean of commercial silk floss (24.0 ± 2.0 mm). In terms of percentage elongation under load, the waxed floss reached 15.0% ± 1.5%. These results indicate that the waxing process confers flexibility and elongation comparable to natural silk, essential properties for maneuvering in the interdental space without breaking.

Flexural and Abrasion Resistance

The Waxed Chambira floss demonstrated a mean flexural resistance of 300 ± 30 folds before breaking, a significant improvement over the untreated fiber (125 ± 15 folds). The abrasion test, which simulates wear from interdental friction, registered a mean resistance of 65 ± 8 frictions. Although these values are lower than those of synthetic floss (200 ± 20 frictions), the abrasion resistance was sufficient to complete cleaning in a typical usage sequence.

Sustainable viability analysis (TBL model)

Economic Viability (Production Cost)

The calculation of the unit production cost for a 10-m segment of Waxed Chambira dental floss resulted in $0.99 USD. The detailed calculation is presented in Appendix B. This cost is considered competitive within the specialized hygiene products market and allows for a reasonable profit margin for local producers.

Social Viability (Public Acceptance)

The descriptive survey applied to a sample of 227 individuals showed high public acceptance: 81.96% of respondents stated they would be willing to try and/or use a natural and ecological dental floss product like Chambira.

Ecological Viability

The ecological assessment confirmed that the collection method used, involving manual and selective defibering of leaves, does not compromise the regeneration or the population of the Chambira palm in its natural habitat, ensuring that production is sustainable in the long term and aligned with conservation goals.

## Discussion

The current investigation sought to validate the technical and sustainable viability of Chambira dental floss by comparing its properties with commercial alternatives. The results confirm the working hypothesis: the Waxed Chambira floss exhibits functional mechanical performance comparable to commercial silk dental floss, establishing itself as a viable ecological alternative for integration into community health projects [9].

The analysis of mechanical properties was crucial in establishing the functionality of the Chambira floss. The waxing process proved to be the key methodological intervention, optimizing the fiber by increasing its tensile strength (maximum stress) by 100% compared to the untreated fiber.

By presenting strength as maximum stress in MPa, a measure is provided that allows for a more rigorous comparison between materials. The Waxed Chambira floss achieved 10.9 ± 0.9 MPa. While this is lower than the performance of synthetic polymers, which often exceed 18 MPa, the obtained value closely approaches the average strength of commercial silk floss 13.7 ± 1.4 MPa). Previous studies on natural fibers have highlighted that their intrinsic strength, although lower than that of industrial polymers, is sufficient for low-load applications, provided their matrix is optimized, as was done with the waxing process [10,11]. The capacity of *A. chambira* fiber to tolerate this stress is consistent with its traditional use as a highly durable textile material [12].

In addition to strength, elasticity and elongation are essential to prevent breakage during interdental use. The Waxed Chambira floss exhibited an elongation of 15.0 ± 1.5%, a value that equated the elongation of silk floss [1]. This result is vital, as the natural wax confers flexibility that compensates for the inherent stiffness of plant fibers, ensuring the floss can maneuver in the gingival sulcus without fraying or premature breakage, overcoming the limitations of the untreated fiber. The superior performance in flexural resistance (300 ± 30 folds) and abrasion resistance (65 ± 8 frictions) confirms that the wax coating mitigates surface wear, a common issue with natural dental flosses [11,13].

The TBL model validated the project across all three dimensions, an increasingly adopted approach in the evaluation of technologies with social impact [14,15]. The 81.96% public acceptance demonstrates a strong demand for ecological products that aligns with growing environmental awareness [7,8]. This high acceptance facilitates the implementation of the product in public health projects, addressing inequality in access to sustainable hygiene solutions [5,6]. Our survey results are consistent with studies that show a growing preference for biomaterials in the healthcare sector, even when the cost is marginally higher [14,15].

The unit cost of $0.99 USD per 10 m suggests a sustainable business model. This price is competitive within the natural segment. The artisanal production model promotes sustainable economic development in Amazonian communities [16,17], establishing an income stream that supports the local circular economy [18]. The articulation of this productive model with the conservation of natural resources exemplifies the sustainable development practices required in the region [19]. Furthermore, conducting an economic viability analysis with a local focus is a recommended practice in the context of product development in Ecuador [20].

The confirmation that the collection method is sustainable resolves a fundamental concern. This directly contrasts with the global problem of pollution from non-biodegradable synthetic polymers that form the basis of most commercial dental flosses [2,3].

Study limitations and future projections

Despite the robustness of the mechanical and viability results, the study presents key limitations that must be acknowledged when interpreting the findings. Due to its in vitro experimental design and limited sample size (*n* = 3), the conclusions are restricted to the descriptive and comparative levels, precluding causal inference regarding clinical efficacy or industrial-scale performance.

The primary constraint is the in vitro nature of the testing. While mechanical tests predict functionality, they do not directly evaluate the biocompatibility, cytotoxicity, or clinical efficacy in plaque removal within the oral cavity. The literature mandates that any material in contact with the mucosa must undergo cytotoxicity and antimicrobial properties testing before clinical use [21,22]. Future research must address these gaps by conducting randomized clinical trials [7], as well as toxicity and pH testing of the waxed material.

An additional methodological limitation is the small experimental sample size (*n *= 3). Although this is an acceptable standard in materials engineering for initial testing, it must be acknowledged that the intrinsic variability of natural fibers is greater than that of synthetic polymers. This necessitates greater statistical rigor, including inferential analysis, in the stages of production scaling to ensure homogeneity and consistent quality.

Future projections are, therefore, centered on: (1) clinical validation and obtaining biological safety certifications, and (2) optimization of the waxing process to reduce diameter variability and increase abrasion resistance, to ensure the consistent quality necessary for large-scale industrial production [22].

## Conclusions

The conclusions are derived directly from the comparative analysis of the mechanical properties and the applied sustainable viability model, aiming to answer the working hypothesis posed in the study. The technical and sustainable viability of artisanal dental floss production based on Chambira palm fiber (*A. chambira*) is confirmed. Treatment with natural wax is fundamental, as it optimizes the fiber's properties, allowing the Waxed Chambira floss to establish itself as an ecological alternative with functional performance.

The Waxed Chambira floss meets the minimum functional requirements for interdental hygiene, validating the working hypothesis. It exhibits elasticity properties (15.0 ± 1.5% elongation) comparable to those of commercial silk floss, and a tensile strength of 10.9 ± 0.9 MPa, which ensures adequate resistance to use and improved handling compared to the untreated fiber. The TBL analysis demonstrated the feasibility of the project across all its dimensions: The unit cost of $0.99 USD per 10 m makes it competitive and profitable for local development. High public acceptance (81.96%) confirms the demand for natural products and supports their integration into community health programs. The selective collection method ensures the regeneration of the Chambira palm, resolving the environmental concern regarding polymer contamination.

In summary, Chambira fiber, when treated with natural wax, represents an effective, biodegradable, and socially responsible solution for public health projects, offering a real alternative to synthetic dental flosses.

## Appendices

Appendix A

**Table 3:** Survey Instrument for Social Viability. The descriptive questionnaire was administered to Dentistry students to determine public acceptance, hygiene habits, and willingness to purchase the organic (Chambira) dental floss. The instrument used to determine social viability was structured around the following variables and included closed-ended questions (dichotomous, multiple-choice, and Likert scale) to capture participant perception.

Key Questions from the Survey Instrument
Knowledge and Habits: How often do you use dental floss in your oral hygiene routine?
Ecological Awareness and Preference: How important is it that personal hygiene products, such as dental floss, are biodegradable or environmentally friendly?
Perception of Effectiveness and Willingness to Pay: Do you consider organic dental floss to be as effective at cleaning teeth as traditional dental floss?

Appendix B: Detailed calculation of unit production cost

**Table 4:** Detailed Calculation of Unit Production Cost This appendix presents the breakdown of the unit production cost for the waxed Chambira dental floss (for 10 meters), which served as the basis for validating economic viability. This calculation justifies the Total Unit Cost of $0.99 USD.

Cost Detail (for 10 meters of floss)	Raw Material ($)	Labor ($)	Indirect Expenses ($)
Chambira Fiber (portion)	$0.20	$0.00	$0.00
Waxing Materials (Natural Cerumen)	$0.35	$0.00	$0.00
Packaging and Labeling	$0.15	$0.00	$0.00
Labor (Defibering and Waxing)	$0.00	$0.25	$0.00
Equipment Depreciation and Energy	$0.00	$0.00	$0.04
Total Unit Cost (10m)	$0.70	$0.25	$0.04
Final Production Cost (USD)			$0.99
